# The respective parts of incidence and lethality in socioeconomic differences in cancer mortality. An analysis of the French network Cancer registries (FRANCIM) data

**DOI:** 10.1186/s12939-019-1087-y

**Published:** 2019-12-03

**Authors:** Joséphine Bryere, Laure Tron, Gwenn Menvielle, Guy Launoy, the French Network of Cancer Registries (FRANCIM), Françoise Galateau-Salle, Anne-Marie Bouvier, Simona Bara, Clarisse Joachim-Contaret, Olivier Ganry, Claire Schvartz, Sandrine Plouvier, Guy Launoy, Emilie Marrer, Patrick Arveux, Pascale Grosclaude, Xavier Troussard, Marc Maynadie, Alain Monnereau, Jean Pierre Daures, Florence Molinie, Anne-Sophie Woronoff, Isabelle Baldi, Jean-Baptiste Nousbbaum, Gaëlle Coureau, Jacqueline Deloumeaux, Marc Colonna, Michel Velten, Tania D’Almeida, Anne-Valérie Guizard, Jacqueline Clavel, Brigitte Lacour, Pierre Ingrand, Sylvie Laumod, Emmanuel Chirpaz, Laure-Manuella Desroziers-Imounga

**Affiliations:** 1 ANTICIPE, Normandie Univ, Unicaen, INSERM, Centre François Baclesse, Avenue du Général Harris, 14076 Caen, France; 2 Sorbonne Université, UPMC Univ Paris 6, INSERM, Institut Pierre Louis d’Epidémiologie et Santé Publique (IPLESP UMRS 1136), 75012 Paris, France

**Keywords:** Cancer, Mortality, Incidence, Lethality, Social inequalities, Attributable deaths

## Abstract

**Background:**

To determine relevant public health actions and to guide intervention priorities, it is of great importance to assess the relative contribution of incidence and lethality to social inequalities in cancer mortality.

**Methods:**

The study population comprised 185,518 cases of cancer diagnosed between 2006 and 2009 recorded in the French registries. Survival was known for each patient (endpoint: 30/06/2013). Deprivation was assessed using the European Deprivation Index. We studied the influence of deprivation on mortality, incidence and lethality rates and quantified the respective proportions of incidence and lethality in social inequalities in mortality by calculating attributable deaths.

**Results:**

For cancers with social inequalities both in incidence and lethality, excess mortality in deprived was mainly caused by social inequalities in incidence (e.g. men lung cancer: 87% of excess deaths in the deprived caused by inequalities in incidence). Proportions were more balanced for some cancer sites (e.g. cervical cancer: 56% incidence, 44% lethality). For cancer sites with a higher incidence in the least deprived (e.g. breast cancer), the excess-lethality in deprived leads entirely the higher mortality among the deprived.

**Conclusions:**

Most of the excess mortality in deprived is due to the excess incidence of tobacco-dependent cancers and the excess lethality of screenable cancers.

## Background

Social inequalities in cancer mortality have been observed worldwide in the last 40 years for most cancer sites [1–5]. This situation has occurred in many countries over time and is even increasing in some [2, 3]. The reduction of social inequalities in health is now a political objective in many countries, including the European Union [6, 7]. It is crucial to elucidate the mechanisms that create health inequalities in order to establish adequate public health policies. Such mechanisms are numerous and have an impact on a wide range of interventions ranging from health promotion to the organization of care and cancer management [8]. With regard to cancer, evidence is accruing that social inequalities affect not only the incidence but also the prognosis of cancers, both of which can contribute to social inequalities in mortality, since the excess deaths of disadvantaged individuals resulting from their greater propensity to develop cancer or/and, independently, from their higher probability of dying because of cancer [9–11].

Several recent studies have attempted to measure social inequalities in incidence, on one hand [1, 12–15] and the social inequalities in lethality, on the other [1, 16–18] and some have tried to quantify the human burden by calculating the excess cases for incidence [19] and the potential gain in life-years for lethality [20, 21]. The latter concerns most of the cancer sites and the prognosis is always worse for the disadvantaged. Such inequalities come from several phenomena: lower participation in screening program, when available, resulting in more cancers diagnosed at an advance stage; and care administered by less specialized institutions and with longer intervention times [16–18].

Large social disparities have been observed for incidence. Unlike lethality, the relationship between socio-economic level and risk of developing cancer is not necessarily unidirectional. For most of the cancer sites, the risk is higher among the deprived (UADT, lung, esophagus, cervix, liver, stomach), while cancer is more frequent for others in the least deprived (breast, prostate, skin) [12–15].

Types of intervention aiming at reducing inequalities in cancer mortality differ according to whether they concern inequalities in incidence or survival thanks to prevention, on one hand, and to the organization of screening and care on the other. From a public health perspective and in order to prioritize interventions, it is thus crucial to evaluate the extent to which the excess lethality and the excess incidence of the different cancer sites contribute to the number of excess deaths in the deprived population. To our knowledge, no study has yet addressed this issue.

The objective of this study was to measure for each cancer site, the proportion of deaths caused by excess incidence and the proportion of deaths caused by excess lethality in the total number of excess deaths among the deprived.

## Materials and methods

### Study population

The study population included all diagnosed cancer cases that were recorded in member registries of the French Network of Cancer Registries (FRANCIM) between 1 January 2006 and 31 December 2009. The population of the area covered by the registries was about 12 million individuals (5,778,595 men and 6,137,751 women), representing 20% of the French population. For the general registries of Gironde and Lille and its region, only cancer cases diagnosed between 1 January 2008 and 31 December 2009 were analyzed (no data were available before this date). For reasons of statistical power, only the 15 most frequent solid tumors were analyzed [22]. The quality and completeness of records of member registries of the FRANCIM network are evaluated every 4 years by the National Committee of Registries. The cancer sites considered were defined according to the codes of the International Classification of Diseases for Oncology, 3rd ed. (ICD-O-3). The study included 185,518 cancer cases.

The reference population came from the Institut National de la Statistique et des Etudes Economiques national census in 2006, 2007, 2008, and 2009. These were given for each IRIS, each sex, and each age group: 15-29, 30-44, 45-59, 60–74, 75 and older. An IRIS is the smallest geographic unit for which census data are available (approximately 2000 individuals with relatively homogeneous social characteristics), an essential factor in this type of study to minimize ecological bias [23]. The study area included 9740 IRIS.

### Data

For all diagnosed cancers, patients’ addresses were geolocalized using Geographic Information Systems (ArcGIS 10.2, ESRI Redlands, California, USA) and assigned to an IRIS. The French version of the European Deprivation Index (EDI) based on the 2007 national census was used to assign a deprivation score to each IRIS. The methodology [24, 25] used an individual deprivation indicator from the conceptual definition of deprivation and selected ecological census variables that are the most closely related to the individual deprivation indicator in the European Union Statistics on Income and Living condition (Euro-SILC). The categorical version (national quintiles) of the EDI was used. 23.77% of the population covered by the cancer registries was in quintile 1 (least deprived), 21.06% in quintile 2, 20.24% in quintile 3, 18.22% in quintile 4, and 16.71% in quintile 5 (most deprived). Recent analyses (not published) showed that the area covered by the French cancer registries is less deprived than the national average for Metropolitan France (using the EDI). In this study, we considered people living in quintile 5 as “deprived” and people living in quintiles 1, 2, 3, 4 as “non-deprived”.

Survival time was available and was calculated as the difference between the date of last information and the date of cancer diagnosis. Follow-up ended on 30 June 2013, i.e. patients alive at that date had their survival time censored. Information on the day or month of the date of last information was missing in less than 0.1% of cases. According to national guidelines, missing days were then replaced by 15 and missing months by July [26]. Loss to follow-up accounted for approximately 2% overall and was censored, also in accordance with national recommendations [26].

### Statistical analysis

The methodology was based on the work done by Camus and Band [27] analyzing the relationship between mortality and cancer incidence in Montréal in Canada between 1984 and 1994. The authors quantified the proportion of the excess mortality in Montréal attributable to the excess incidence and attributable to the excess lethality compared to the rest of the country (excluding Quebec). We adapted the method to our data.

**Fig. 1:**
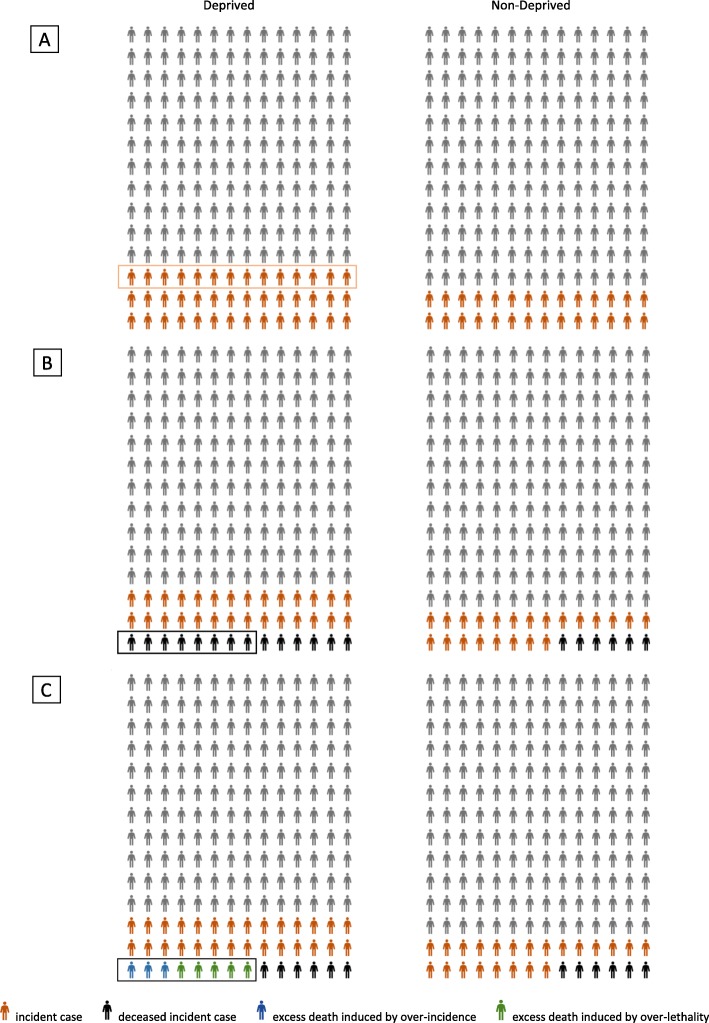
Statistical methodology used

The methodology is illustrated in Fig. 1 with an example where incidence and lethality are greater for the deprived (proportions not representative of reality). For each IRIS, the number of person-years per million inhabitants was calculated. The IRIS were classified as “deprived” and “non-deprived” using the quintiles of EDI as described in the data subsection. The number of incident cancer cases by sex, age group and by IRIS was known for each cancer site. Thus, we identified the incident cases in these two populations (in red in Fig. 1_a). We performed a Poisson regression (after checking over-dispersion) to estimate the incidence rate of deprived (*IR*_*deprived*_) and the relative risk of incidence of deprived versus non-deprived (*RR*_*incidence*_), from which the number of excess cancers per year and per million inhabitants in the deprived was deduced (framed in red in Fig. 1_a, ${ExcessCases}_{deprived}=\left({IR}_{deprived}\times 1.{10}^6\right)-\left(\frac{IR_{deprived}\times 1.{10}^6}{RR_{incidence}}\right)$).

Next (Fig. 1_b), we identified deaths among cancer cases (in black in Fig. 1_b). An incident case was considered dead if the death occurred within 5 years of diagnosis. We also performed a Poisson regression (after checking over-dispersion) to estimate the mortality rate of the deprived (*MR*_*deprived*_) and the relative risk of mortality of the deprived versus the non-deprived (*RR*_*mortality*_), from which the number of excess deaths in the deprived was deduced in the same way as for incidence (framed in black in Fig. 1_b, ${ExcessDeaths}_{deprived}=\left({MR}_{deprived}\times 1.{10}^6\right)-\left(\frac{MR_{deprived}\times 1.{10}^6}{RR_{mortaity}}\right)$).

The final step (Fig. 1_c) consisted in quantifying the excess deaths that were attributable to the excess incidence and those attributable to the excess lethality. We calculated the lethality rate of the non-deprived by dividing the number of deaths by the number of incident cancer cases in this population (*LR*_*non* − *deprived*_). We multiplied the number of excess cancer cases among the deprived (*ExcessCases*_*deprived*_, corresponding to red individuals framed in Fig. 1_a) by the lethality rate of the non-deprived considered as the reference population (*LR*_*non* − *deprived*_). Thus, we obtained the theoretical number of excess deaths among the deprived per year and per million inhabitants resulting solely from the excess incidence among the deprived independently of the different lethality rates between the deprived and the non-deprived, since we applied the lethality rate of the non-deprived (reference population) to the deprived (in blue in Fig. 1_c *ExcessDeathsIncidence*_*deprived*_ = *ExcessCases*_*deprived*_ × *LR*_*non* − *deprived*_). We deduced excess deaths due to excess lethality by subtracting excess deaths due to over-incidence from total excess deaths (in green in Fig. 1_c, *ExcessDeathsLethality*_*deprived*_ = *ExcessDeaths*_*deprived*_ − *ExcessDeathsIncidence*_*deprived*_). Finally, this allowed us to calculate the percentage of excess deaths induced by social inequalities in incidence and the percentage of excess deaths induced by social inequalities in lethality.

All analyzes were adjusted by age group and stratified by sex and were performed using SAS software version 9.4.

## Results

A total of 185,518 cancer cases were analyzed, with 107,780 in men and 77,738 in women (Table 1). Among the incident cases, 79,461 patients (51,854 men and 79,461 women) were considered dead meaning that their death occurred within 5 years after diagnosis.

**Table 1:** Site definition and frequencies in member registries of the FRANCIM network between 2006 and 2009

Site	ICD-O-3^a^	Morphology	Men	Frequencies				
	Topography				Women		Total	
			Incidence	Deaths^b^	Incidence	Deaths^b^	Incidence	Deaths^b^
Colon-Rectum	C18, C19, C20, C21	All	16,339	7732	13,348	6101	29,687	13,833
Cervix	C53	All			1843	681	1843	1
Uterus	C54	All			4121	1253	4121	1253
Stomach	C16	All	3493	2734	1905	1389	5398	4123
Liver	C22	All	4979	4248	1115	943	6094	5191
Larynx	C32	All	1881	881			1881	881
Lips-Mouth-Pharynx	C0, C10, C11, C12, C13, C14	All	5766	3629	1406	704	7172	4333
Melanoma	C44	87,203–87,803	2694	584	3158	430	5852	1014
		97,603–97,643						
Esophagus	C15	All	3250	2784			3250	2784
Ovary	C56	All excluding			2966	1682	2966	1682
		{84,423; 84,513;						
		84,613; 84,623;						
		84,723; 84,733}						
Pancreas	C25	All	3416	3136	3187	2949	6603	6085
Lung	C33, C34	All	16,248	13,802	4964	3984	21,212	17,786
Prostate	C61	All	36,585	6422			36,585	6422
Kidney	C64, C65, C66	All	3704	1326	1933	617	5637	1943
Breast	C50	All			31,787	4930	31,787	4930
Central nervous system	C70, C71, C72	≤ 91,103 or ≤ 91,800	1681	1363	1318	1016	2999	2379
		99,833,99,853,						
		99,863,99,873,						
		99,893						
Testis	C62	All	1332	75			1332	75
Thyroid	C73	All	1081	129	3407	140	4488	269
Bladder	C67	All	5331	3009	1280	788	6611	3797

^a^ ICD-O-3: International Classification of Diseases for Oncology, 3rd Edition

^b^ An incident case was considered dead if the death occurred within 5 years of diagnosis

**Fig. 2:**
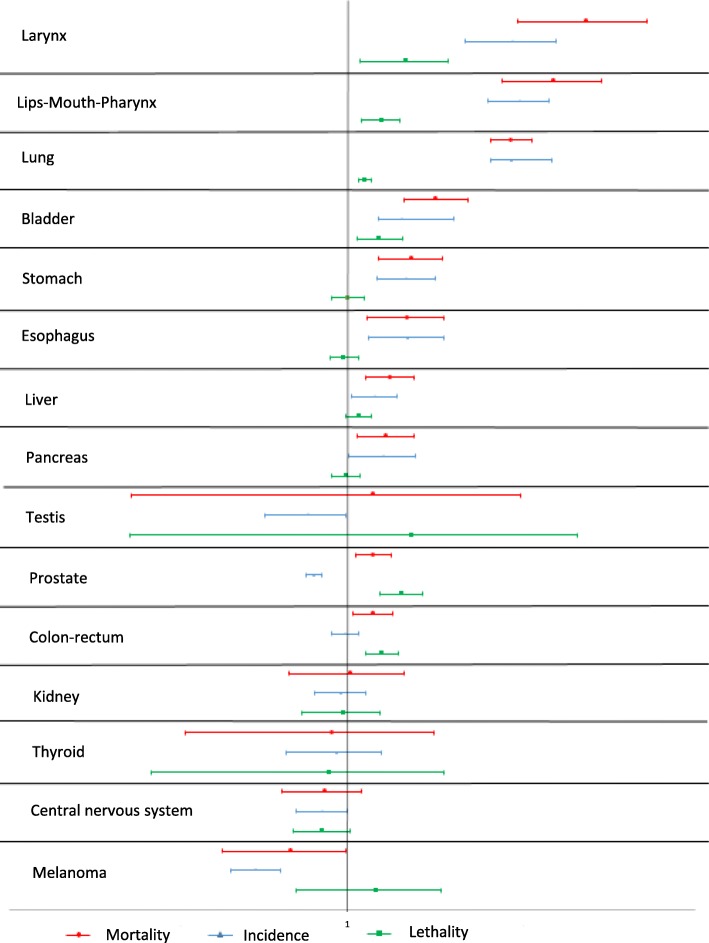
Relative risks and confidence intervals (95%) of mortality, incidence and lethality of deprived versus non-deprived in men

**Fig. 3:**
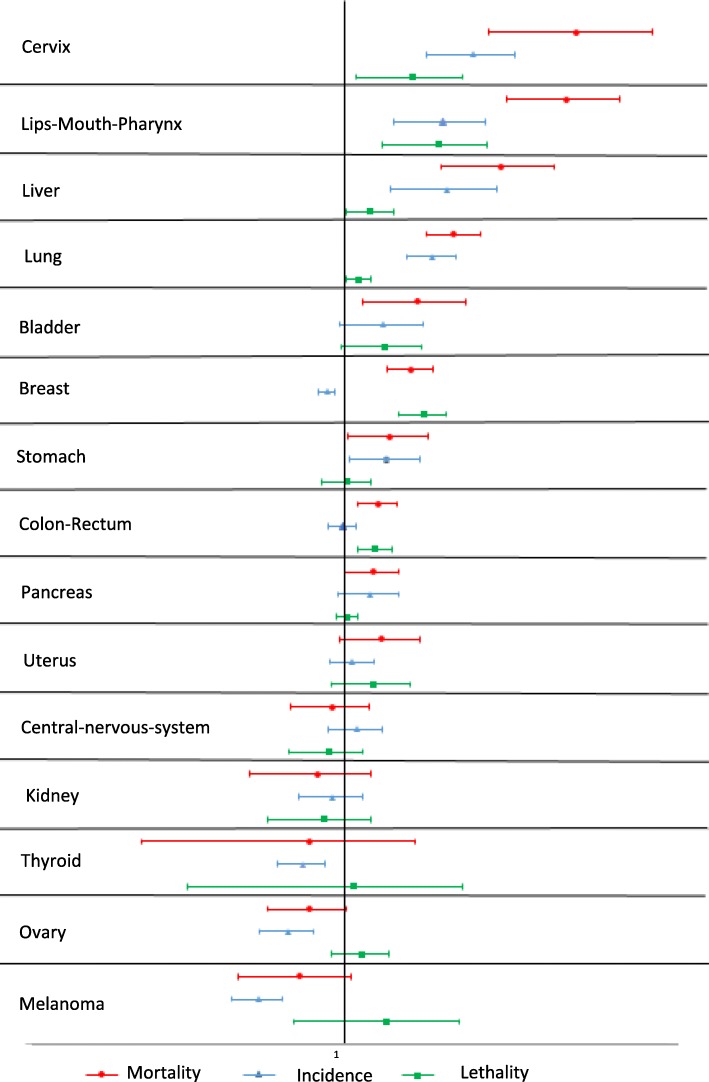
Relative risks and confidence intervals (95%) of mortality, incidence and lethality of deprived versus non-deprived in women

Figures 2 and 3 show the relative risk of mortality, incidence and lethality of the deprived versus the non-deprived for each cancer site. All the successive elements used for the calculation of the final proportions are provided in Additional file 1:Table S1 and Additional file 2: Table S2. For men (Fig. 2), mortality was significantly higher among the deprived for larynx, lips-mouth-pharynx, lung, bladder (with incidence and lethality significantly higher among the deprived with relative risks if incidence higher than relative risks of lethality), stomach, esophagus (only incidence significantly higher among the deprived), liver, pancreas (neither incidence nor lethality significantly associated with deprivation), prostate (incidence inversely significantly associated with deprivation whereas lethality significantly higher among the deprived) and colon-rectum (only lethality associated with deprivation).

For women (Fig. 3), the mortality was significantly higher among the deprived for cervix, lips-mouth-pharynx (incidence and lethality both significantly higher among the deprived), liver, lung (only incidence significantly associated with deprivation), bladder (neither incidence nor lethality associated with deprivation), breast (incidence significantly higher among the non-deprived whereas lethality significantly higher among the deprived) and colorectal (only lethality was significantly associated with deprivation) cancers.

**Fig. 4:**
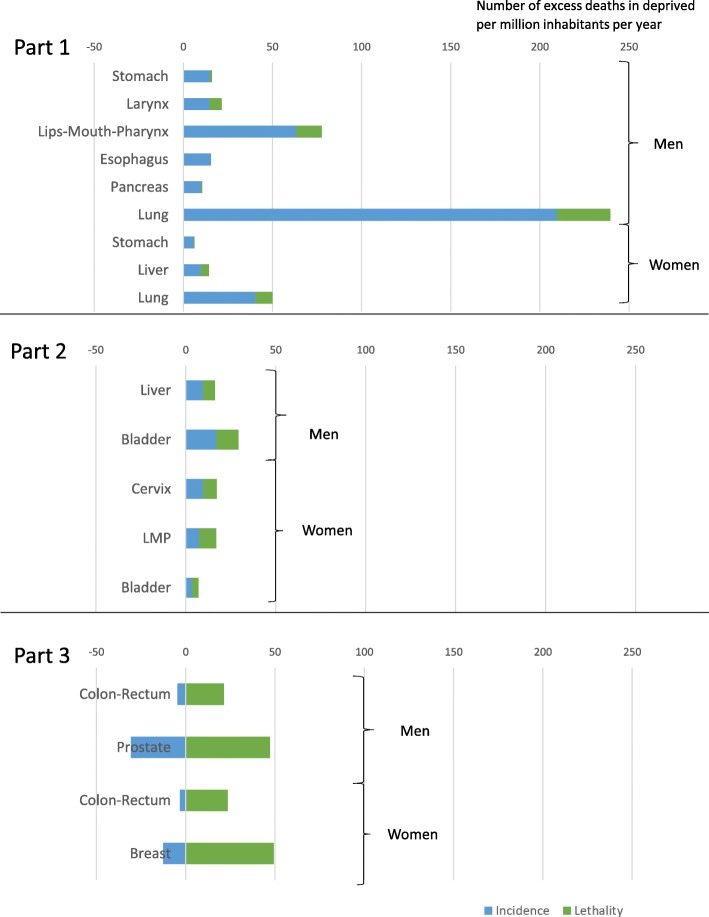
Respective proportion of deaths induced by social inequalities in incidence and lethality in social inequalities in cancer mortality. Part 1: Excess deaths mainly caused by inequalities in incidence, Part2: Excess deaths shared by inequalities in incidence and lethality, Part 3: Excess deaths only caused by inequalities in lethality with an opposite association of incidence

Figures 4 presents the number of excess deaths in the deprived per million inhabitants per year for three different patterns, with excess deaths attributable to the excess incidence and those attributable to the excess lethality. Only cancer sites with a significant association between mortality and deprivation are presented.

The first pattern (Fig. 4_part 1) includes cancer sites with a higher incidence and lethality among the deprived and with excess deaths that are mainly caused by social inequalities in incidence. For men, the respective proportions of excess deaths caused by incidence and lethality were 95–5% for stomach cancer, 68–32% for larynx cancer, 82–18% for lips-mouth-pharynx cancer, 100–0% for esophageal cancer, 95–5% for pancreatic cancer and 95–5% for lung cancer. Each year, per 1 million inhabitants, more than 200 deaths in excess in deprived males were due to the excess incidence in the deprived of lung cancer. For women, the respective proportions were 92–8% for stomach cancer, 69–31% for liver cancer and 81–19% for lung cancer.

The second pattern (Fig. 4_part 2) includes cancer sites with a higher incidence and lethality among the deprived but with more shared proportions. For men, the respective proportions of excess deaths caused by incidence and lethality were 60–40% for liver cancer and 58–42% for bladder cancer. For women, the respective proportions were 56–44% for cervical cancer, 44–56% for lips-mouth pharynx cancer and 50–50% for bladder cancer.

The third pattern (Fig. 4_part 3) concerns cancer sites with a higher incidence among affluent people, for which the mortality remained significantly higher in the deprived because of excess deaths due to the excess lethality in the deprived. For each cancer site, colon-rectum in both sexes, prostate in males and breast in females, the excess lethality in deprived people was higher than the reduced number of cases in the deprived.

## Discussion

Thanks to an innovative methodology, this study is the first to evaluate the share due to social inequalities of incidence and social inequalities of lethality in the social inequalities of cancer mortality. We were able to evaluate for each cancer site the number of excess deaths associated with deprivation and attributable to either the excess incidence or the excess lethality. Our results show that the underlying mechanisms of social inequalities in cancer mortality are different depending on the cancer site and that the fight against these inequalities must be organized differently depending on the cancer.

Three different patterns were identified depending on the relative contribution of the excess incidence or the excess lethality in excess deaths among the deprived. The first pattern comprises cancer sites with higher incidence and lethality among the deprived and for which social inequalities in mortality were mainly due to social inequalities in incidence. It mainly concerns tobacco-related cancers and both tobacco- and alcohol-related cancers such as head and neck, lung and digestive cancers like esophageal and stomach cancer in both sexes and liver cancer in females. These risk factors are known to be socially stratified with an over-representation in the deprived [28]. A study conducted in France [29], reported that increasing levels of deprivation were associated with a greater likelihood of tobacco use and alcohol use, with odds ratios reaching 1.93 and 1.80 in the more deprived compared with the less deprived for tobacco and alcohol, respectively. The result obtained in the present study may also be explained by the fact that survival is very low for these cancer sites, regardless of socioeconomic status. The latest French estimates [26] report a 5-years net survival equal of 17% for lung cancer and equal to 37% for UADT cancer, which places them among the cancers with the worst prognosis. Thus, the probability of survival is very low regardless of socioeconomic status, so this also explains why most of the excess deaths are attributable to the excess incidence among the deprived. This group consists mainly of men in majority. From a public health point of view, the major contribution of excess death in the deprived in this group is due to the excess incidence of lung cancer and head and neck cancer in men. Cancer trends analyses suggest that the situation will be similar for women in the coming years [30–33]. A Spanish study predicted an annual increase in incidence to 7.0% in oral cavity cancers and of 3.1% in lung cancers for women [34]. Therefore, the repercussions on the number of excess deaths among the deprived concerning these cancer sites could be of great importance.

The second pattern includes cancer sites where the contribution of the excess incidence and the excess lethality are of comparable. Both for men and women, this concerns the bladder in both sexes, the liver in males, and the lip-mouth-pharynx and cervix in females. It would seem that these cancer sites do not require special attention with regard to social inequalities in incidence or lethality. Among these cancer sites, the cervix is the only one for which there is a screening test known to reduce both incidence and mortality [35, 36]. There was no nationwide organized program implemented at the time of the study. Since uptake of this screening is socially stratified [37], reducing this social gradient could have an impact at all stages of the construction of social inequalities in mortality related to cervical cancer.

The third pattern, i.e. the reduced incidence and the excess lethality in the deprived, concerns cancer sites for which screening is performed in France, i.e. organized screening for breast and colorectal cancer and non-organized screening for prostate cancer. For these cancer sites, both the reduced incidence and the excess lethality can be partly explained by the lower participation in screening among the deprived reported in numerous papers on organized screening [38, 39]. For example, in colorectal cancer, a 24% difference in participation between the least and the most deprived was observed [35]. The odds ratio was 0.71 in the most deprived compared to the least deprived in mammography screening programs [39]. The under-incidence in the deprived (or over-incidence in affluent people) is due to the inevitable contribution of over-diagnosis associated with screening [40–43] which is particularly the case for prostate cancer [40, 43]. The proportion of over-diagnosis was 10-14% for breast cancer [42] and as high as 67% for prostate cancer [43]. However, participation in the screening programs is not the only explanation for the incidence results. For breast cancer in particular, the excess incidence among the affluent can be partly explained by the social distribution of some risk factors, such as age at birth of first child or breastfeeding. For colorectal cancer, social disparities in screening participation were demonstrated in the literature. However, no association between deprivation and incidence was observed in our data. Our results on lethality, though, may reflect such disparities in screening. This inconsistency may result from the fact that social differences in screening are too small to be observed on the incidence or that some risk factors are more frequent in the disadvantaged population such as poorer diet, which rebalances incidence rates. The excess lethality in the deprived for prostate cancer is mainly due to a delay in diagnosis. Furthermore, the cancer sites concerned have a very high survival compared to other cancer sites. The latest French estimates [26] report a 5-years net survival of 94% for prostate cancer, to 88% for breast cancer and to 60% for colorectal cancer. We thus suppose that social inequalities have more time to become apparent for these cancer sites during the care path, which results in a greater importance on social inequalities in mortality. Due to the high frequency of these cancers, the number of excess deaths in the deprived people due to the excess lethality for these screenable cancer sites is significant from a public health point of view.

We decided to calculate the excess deaths attributable to incidence and lethality only for cancer sites with a significant association between deprivation and mortality. Some cancer sites have not been studied in detail but had associations at the limit of significance such as ovary and melanoma. For these cancer sites, the upper bound of the confidence interval was very close to the value 1 suggesting a higher mortality in affluent populations, which is a result that was unobserved so far. This may result from a much higher proportion of cancer diagnoses in the affluent (possibly resulting from social differences in screening participation for melanoma and in the distribution of reproductive risk factors for ovarian cancer), non-compensated, this time, by social inequalities in lethality.

Our study has some limitations. Time between diagnosis and death was not considered because we were unable to do so. The cause of death was not available in the registry data so other causes of deaths were not excluded from the analyses. It would have been interesting to include only cancer-related deaths in our study. Other issues are worthy of attention. Firstly, although our study population included all cases of cancer recorded in French registries between 2006 and 2009, this represented only 20% of the French population. Paris, Marseille and Lyon, the three largest cities in France, are not covered by the registries, and the study area is not representative of the national territory for deprivation. Indeed, registries in France are defined at the local level and a national registry does not exist. Secondly, our study design used aggregated data at the IRIS level, with an ecological index of deprivation. Although this type of index presents many advantages such as applicability to all populations, thus preventing selection bias, its main weakness is unavoidable ecological bias in measuring the social deprivation of individuals. The same score is assigned to all people living in the same area. However, this bias is limited by the use of IRIS, which is the smallest geographic level with available census data. Finally, since no other studies of this type are available in the current literature, we cannot compare our results with others. However, the results concerning the social inequalities in incidence and lethality separately are however in agreement with the available studies, suggesting the coherence of our observations.

The excess mortality by cancer in the deprived is shared by all cancer sites and a wide range of mechanisms are involved in these inequalities that accrue throughout the care path of a patient. However, from a public health point of view and to rationalize public policies aimed at reducing these social inequalities, our results show that these inequalities are major for preventable or detectable and screenable cancers, and that most of the excess mortality is due to the excess incidence of tobacco-dependent cancers and the excess lethality of screenable cancers. Policies that promote smoking cessation in deprived populations and reducing the social inequalities in screening participation are certainly those that are best able to tackle social inequalities in mortality in the field of cancer.

## Supplementary information

**Additional file 1: Table S1.** Successive elements needed for the calculation of the number of excess deaths in deprived attributable to excess incidence and excess lethality per million inhabitants per year in men (FRANCIM data between 2006 and 2009)

**Additional file 2: Table S2.** Successive elements needed for the calculation of the number of excess deaths in deprived attributable to excess incidence and excess lethality per million inhabitants per year in women (FRANCIM data between 2006 and 2009)

## Data Availability

The data that support the findings of this study came from the FRANCIM network, but restrictions apply to the availability of these data, which were used under license for the current study, and so are not publicly available.
