# Supplementary material for: The respective parts of incidence and lethality in socioeconomic differences in cancer mortality. An analysis of the French network Cancer registries (FRANCIM) data
Source: Int J Equity Health. 2019 Dec 3;18:189. doi: 10.1186/s12939-019-1087-y (PMC6891983; doi:10.1186/s12939-019-1087-y)
Supplement: Supplementary file 2 — Additional file 2: Table S2. Successive elements needed for the calculation of the number of excess deaths in deprived attributable to excess incidence and excess lethality per million inhabitants per year in women (FRANCIM data between 2006 and 2009) [file 12939_2019_1087_MOESM2_ESM.docx]

**Supplementary table 2 : Successive elements needed for the calculation of the number of excess deaths in deprived attributable to excess incidence and excess lethality per million inhabitants per year in women (FRANCIM data between 2006 and 2009)**

|  | *First step (corresponding to figure 1_A)* | | | *Second step (corresponding to figure 1_B)* | | | *Third step (corresponding to figure 1_C)* | | |
| --- | --- | --- | --- | --- | --- | --- | --- | --- | --- |
| **Cancer site** | $\boldsymbol{IR}_{\boldsymbol{deprived}}$ | $\boldsymbol{RR}_{\boldsymbol{incidence}}$ ***(95% CI)*** | $\boldsymbol{ExcessCases}_{\boldsymbol{deprived}}$ | $\boldsymbol{MR}_{\boldsymbol{deprived}}$ | $\boldsymbol{RR}_{\boldsymbol{mortality}}$ ***(95% CI)*** | $\boldsymbol{ExcessDeaths}_{\boldsymbol{deprived}}$ | $\boldsymbol{LR}_{\boldsymbol{non-deprived}}$ | $\boldsymbol{ExcessDeathsIncidence}_{\boldsymbol{deprived}}$ **(percentage )** | $\boldsymbol{ExcessDeathsLethality}_{\boldsymbol{deprived}}$ **(percentage )** |
| Colon-Rectum | 0.00040 | 0.98 (0.94 - 1.03) | -7.49 | 0.00023 | 1.09 (1.04 - 1.06) | 20.21 | 0.44 | -3.31 (-16%) | 23.52 (116%) |
| Cervix | 0.00011 | 1.39 (1.26 - 1.53) | 30.52 | 0.00004 | 1.71 (1.48 - 1.99) | 17.20 | 0.32 | 9.65 (56%) | 7.55 (44%) |
| Uterus | 0.00018 | 1.02 (0.95 - 1.09) | 2.79 | 0.00006 | 1.11 (0.99 - 1.24) | 5.48 | 0.30 | 0.82 (15%) | 4.65 (85%) |
| Stomach | 0.00008 | 1.12 (1.02 - 1.24) | 8.26 | 0.00005 | 1.13 (1.01 - 1.26) | 6.30 | 0.70 | 5.79 (92%) | 0.51 (8%) |
| Liver | 0.00005 | 1.31 (1.16 - 1.48) | 11.87 | 0.00004 | 1.48 (1.31 - 1.66) | 14.36 | 0.83 | 9.84 (69%) | 4.51 (31%) |
| Lips-Mouth-Pharynx | 0.00008 | 1.29 (1.16 - 1.44) | 18.73 | 0.00004 | 1.68 (1.46 - 1.93) | 16.95 | 0.40 | 7.44 (44%) | 9.51 (56%) |
| Melanoma | 0.00011 | 0.72 (0.65 - 0.81) | -40.69 | 0.00002 | 0.85 (0.70 - 1.04) | -2.78 | 0.14 | -5.51 (198%) | 2.73 (-98%) |
| Ovary | 0.00010 | 0.82 (0.74 - 0.91) | -23.55 | 0.00006 | 0.88 (0.77 - 1.01) | -8.40 | 0.58 | -13.60 (162%) | 5.21 (-62%) |
| Pancreas | 0.00012 | 1.07 (0.98 - 1.17) | 7.95 | 0.00011 | 1.08 (0.99 - 1.17) | 8.09 | 0.92 | 7.29 (90%) | 0.81 (10%) |
| Lung | 0.00025 | 1.26 (1.19 - 1.34) | 51.34 | 0.00020 | 1.33 (1.25 - 1.42) | 50.00 | 0.79 | 40.46 (81%) | 9.54 (19%) |
| Kidney | 0.00009 | 0.95 (0.86 - 1.06) | -4.54 | 0.00002 | 0.90 (0.73 - 1.11) | -2.53 | 0.29 | -1.29 (51%) | -1.23 (49%) |
| Breast | 0.00121 | 0.94 (0.91 - 0.96) | -83.53 | 0.00023 | 1.19 (1.13 - 1.27) | 36.76 | 0.15 | -12.57 (-34%) | 49.33 (134%) |
| Central nervous system | 0.00012 | 1.03 (0.95 - 1.12) | 3.33 | 0.00004 | 0.95 (0.84 - 1.08) | -2.00 | 0.35 | 1.15 (-58%) | -3.14 (158%) |
| Thyroid | 0.000129 | 0.86 (0.79 - 0.94) | -21.00 | 0.000005 | 0.88 (0.56 - 1.40) | -0.70 | 0.04 | -0.85 (121%) | 0.15 (21%) |
| Bladder | 0.00006 | 1.11 (0.99 - 1.25) | 6.52 | 0.00004 | 1.22 (1.07 - 1.39) | 7.01 | 0.51 | 3.53 (50%) | 3.47 (50%) |
